# Calculated initial parenteral treatment of bacterial infections: Safety and tolerabilty

**DOI:** 10.3205/id000060

**Published:** 2020-03-26

**Authors:** Ralf Stahlmann, Hartmut Lode

**Affiliations:** 1 Institut für Klinische Pharmakologie und Toxikologie, Charité – Universitätsmedizin Berlin, Germany; 2 Research Center Charlottenburg, Berlin, Germany

## Abstract

This is the fourth chapter of the guideline “Calculated initial parenteral treatment of bacterial infections in adults – update 2018” in the 2^nd^ updated version. The German guideline by the Paul-Ehrlich-Gesellschaft für Chemotherapie e.V. (PEG) has been translated to address an international audience.

Safety and tolerability of antimicrobial agents will be discussed in this chapter. Toxic, allergic and biological effects can be differentiated on the basis of their pathogenesis. The question of differences in the tolerability of specific antibiotics is of particular importance. However, due to limitations of the available data, it cannot be answered for most agents with the desired accuracy. For an assessment of rare side effects, results from the postmarketing surveillance have to be used.

## Zusammenfassung

Dies ist das vierte Kapitel der von der Paul-Ehrlich-Gesellschaft für Chemotherapie e.V. (PEG) herausgegebenen S2k Leitlinie „Kalkulierte parenterale Initialtherapie bakterieller Erkrankungen bei Erwachsenen – Update 2018“ in der 2. aktualisierten Fassung.

Die Sicherheit und Verträglichkeit von antimikrobiellen Wirkstoffen werden in diesem Kapitel diskutiert. Toxische, allergische und biologische Wirkungen können auf der Basis ihrer Pathogenese unterschieden werden. Die Frage nach Unterschieden in der Verträglichkeit verschiedener Antibiotika ist von besonderer Bedeutung. Sie kann jedoch häufig nicht mit der gewünschten Genauigkeit beantwortet werden, weil die verfügbaren Daten limitiert sind. Für eine Bewertung seltener Nebenwirkungen müssen die Ergebnisse aus der Überwachung nach der Zulassung herangezogen werden.

## Introduction

Adverse effects must be expected in about 10% of treated patients. This is true for most parenteral antibiotics. In some cases, the frequency of adverse drug reactions is higher. The question of differences in the tolerability of the available preparations is of particular importance – but often cannot be answered with the desired accuracy due to a lack of studies or inadequate studies. It is not justified to assess differences in drug tolerance by direct comparison of the results from different clinical trials. 

Despite extensive standardization of clinical trials, only data from comparative studies that are preferably double-blind studies, provide a reliable source for direct comparisons between different drugs. This applies to the undesirable effects as well as to the desired effects. The number of patients enrolled in comparative clinical trials is insufficient to predict rare adverse effects. Therefore, further analysis of the overall data from multiple clinical trials or even the experiences from postmarketing surveillance have to be used. However, the limitations of such data should always be considered.

In general, the adverse effects of most parenterally administered anti-infective agents are manifested predominantly in three organ systems:

- gastrointestinal tract (e.g. nausea, vomiting, diarrhea),
- skin (e.g. rash, urticaria, phototoxicity) and
- CNS (e.g. headache, dizziness, sleep disorders).

Significant differences exist in the severity and frequency of a particular adverse effect. Toxic, allergic and biological effects can be differentiated based on the pathogenesis of the undesirable effects. In some cases it cannot be determined clearly whether gastrointestinal disorders for instance were caused by direct effects on the corresponding organs or whether the changes are caused by an impact on the bacterial flora.

It should be remembered that every dose of an antimicrobial substance affects the body’s own flora. For each antibacterial treatment, therefore, the biological side-effects of the substances must be taken into account in the benefit/risk assessment. The nature and extent of the changes are significantly influenced by the antibacterial effect and the pharmacokinetic properties of the antibiotic. Antibiotic-associated colitis is triggered by toxin-producing strains of *Clostridium* *difficile*. This serious adverse effect is becoming increasingly common and is apparently often overlooked. According to an estimate, each year about 40,000 hospitalized patients in Europe suffer from undetected *Clostridium* *difficile* infections due to lack of clinical inquiry and sub-optimal diagnosis [1].

The following definitions are used in specifying the frequencies of adverse effects: “very common” if the adverse effect is expected in more than 10% of patients, an incidence between >1% and 10% is referred to as “frequent”, between >0.1% and 1% as “occasional”. An adverse effect is classified as “rare” if the frequency is between >0.01% and 0.1%; if it occurs in less than one patient per 10,000, it is considered “very rare”. 

## Drug-specific side effects

### Beta-lactam antibiotics

Parenterally administered beta-lactam antibiotics are generally well tolerated. The side effects are usually mild and transient and rarely require premature discontinuation of treatment. Hypersensitivity reactions in the form of morbilliform or scarlatiniform exanthema can occur in about 1–2% of patients. Rarely (in 0.5–1%) swelling of the face, tongue or glottis occurs (e.g. Quincke’s edema). Pneumonitis, interstitial pneumonia or interstitial nephritis are very rare. Severe acute allergic reactions (anaphylaxis or even life-threatening shock) occur rarely. These can be triggered even by low single doses and generally can occur up to 30 minutes after application. Such reactions are more common after penicillins than other beta-lactam antibiotics. Patients should be observed for approximately 30 minutes after administration of a beta-lactam antibiotic to detect any possible allergic reaction.

Cross-allergic reactions between penicillins and cephalosporins are rather rare. Aztreonam is a beta-lactam antibiotic that can also be used in patients who developed rashes or other types of acute hypersensitivity reactions to penicillins and other beta-lactams because, according to previous experience, cross-allergies are very rare. In some patients, allergic reactions have been observed under aztreonam treatment, but these are likely related to the structure of the side chain rather than the beta-lactam ring. Since the side chain is identical to the corresponding structure in ceftazidime, the monobactam aztreonam should not be administered in an allergic reaction to ceftazidime and vice versa [2], [3].

Effects on the blood count are allergic or toxic. They occasionally manifest in the form of thrombocytopenia and/or eosinophilia. In particular in cases of long-term administration, blood count checks are indicated at regular intervals, since it can lead to leukopenia and neutropenia. The changes are generally reversible within a few days after discontinuation of the antibiotic.

Gastrointestinal intolerance symptoms such as loss of appetite, nausea, vomiting, abdominal pain, meteorism, or soft stools are commonly observed during treatment with beta-lactam antibiotics. Diarrhea (passing more than 3 unformed stools per day) occurs in 2–10% of patients.

When infused with ceftobiprole, gastrointestinal disturbances, such as nausea and vomiting, were dependent on infusion duration. Frequently, patients reported changes to their sense of taste during the clinical trial of the substance [4].

Reversible, moderate changes in liver function parameters (such as transaminases, alkaline phosphatase) occur in up to 10% of patients. In isolated cases, transient cholestatic hepatitis has been observed. The risk is increased the older the patient or the more prolonged the use. Amoxicillin/clavulanic acid should only be used in elderly patients (>65 years) under liver function control.

Detailed knowledge is now available on the mechanism of flucloxacillin-induced liver damage. The incidence was about 1 in 12,000 patients who received the drug for the first time. In vitro it was shown that – in contrast to flucloxacillin itself – the 5'-hydroxylmethyl metabolite has a more toxic effect on biliary epithelial cells than on hepatocytes. A very clear association also exists with the HLA allele B*5701, from which it can be deduced that faulty T-cell activity in the pathogenetic process is also jointly responsible. The odds ratio here was around 80 [5].

High dose intravenous treatment with amoxicillin can lead to crystalluria and kidney failure. Adequate fluid intake and alkalization of the urine can prevent crystallization of the antibiotic [6].

In ceftriaxone treatment, in rare cases, shadows were observed in the sonogram of the gallbladder, which disappeared after discontinuation or at the end of treatment (so-called transitory biliary pseudolithiasis).

In certain risk constellations (patients with severe renal impairment, epilepsy, impairment of the blood-brain barrier, such as meningitis) and cases of administration of beta-lactam antibiotics at very high doses, seizures may occur. The seizure-causing potential of newer carbapenems was shown in animal testing to be lower than that of imipenem/cilastatin. Therefore, in contrast to imipenem/cilastatin, meropenem is also approved for the treatment of meningitis [7].

Administration of meropenem or other carbapenems together with valproic acid significantly reduces plasma levels of the anti-epileptic drug and can cause seizures. Valproic acid is mainly metabolized through glucuronidation. The metabolite is then partially but still hydrolytically, split again. Apparently, carbapenems inhibit the hydrolysis of glucuronide and thus result in there being less free valproic acid in the plasma. The serum levels of valproic acid must therefore be controlled and the dosage possibly adjusted when a carbapenem is administered concomitantly [8].

Long-term and repeated use of beta-lactam antibiotics (especially those with a broad antibacterial spectrum) may result in superinfection or colonization with resistant, potentially pathogenic agents such as *Clostridium* *difficile* or budding yeasts (e.g. oral thrush, vulvovaginitis).

### Fluoroquinolones

During treatment with fluoroquinolones (for example ciprofloxacin, levofloxacin, moxifloxacin), adverse events occur in approximately 4–10% of treated patients [9], [10]. 

Most commonly, undesirable effects manifest themselves in the gastrointestinal tract or CNS (e.g. insomnia, drowsiness, occasionally psychotic reactions and seizures). As regards skin reactions, the phototoxic potential of fluoroquinolones has received especial attention. In fluoroquinolone treatment direct exposure to UV light should be avoided; the phototoxic potential of the three quinolones which can be administered parenterally is relatively low. Cardiotoxic effects were initially observed in animal testing after administration of fluoroquinolones that are no longer common, such as sparfloxacin. Minor changes in QTc time can also occur in humans. Fluoroquinolones may not be combined with other arrhythmogenic medicinal products. Also quinolones should only be used after a careful risk-benefit assessment [11] in patients with Long QT syndrome, uncorrected electrolyte imbalances (hypokalaemia and hypomagnesemia), as well as heart disease, such as heart failure, myocardial infarction, bradycardia. 

Although fluoroquinolones are contraindicated in children, adolescents and pregnant women, the clinical relevance of fluoroquinolone-specific toxic effects on immature articular cartilage is controversial. Ciprofloxacin can be used to treat severe infections in children and adolescents when deemed necessary. It has been used for a long time in juvenile cystic fibrosis patients with *Pseudomonas*-induced bronchopulmonary infections, without causing an increase of joint complaints. In addition, it is approved for this age group for complicated urinary tract infections and pyelonephritis and after inhalation of anthrax pathogens for post-exposure prophylaxis and treatment. 

Inflammation or rupture of the Achilles tendon may occur as rare adverse effects after each fluoroquinolone. This undesirable effect is more common in people over the age of 60 but younger patients are also affected. Since 2008 this side effect has been indicated in the USA with a “black box warning” [12].

In 2013 the US Food and Drug Administration (FDA) noted the risk of peripheral neuropathy through quinolones. They can occur even after short treatment and may be irreversible. According to the European “Information for Professionals”, treatment with a quinolone should be stopped immediately if patients develop symptoms such as pain, burning, tingling, numbness or weakness.

In very rare instances, cases of hepatitis to liver failure have been associated with administration of fluoroquinolones.

In 2013 one study found a clear association between treatment with a quinolone and retinal detachment. In further, more recent work, however, such a connection was not confirmed [13]. 

Among the fluoroquinolones currently available for parenteral therapy, ciprofloxacin in particular inhibits cytochrome P450-1A2. This delays the breakdown of theophylline and caffeine but also the metabolism of other drugs, for example clozapine, can be inhibited to a clinically relevant extent [14].

Due to the partly severe adverse effects, the EMA has initiated a reassessment of the benefit-risk balance of fluoroquinolones (see chapter 1 [15]).

### Macrolides, azalides

In addition to erythromycin, clarithromycin and azithromycin are also available for parenteral therapy. Because of the divergent basic structure, azithromycin is also referred to as “azalide”. Macrolides/azalides often lead to local incompatibility reactions at the infusion site.

Apart from the local intolerance reactions, the most common adverse effects of these antibiotics – including by parenteral administration – are gastrointestinal disorders. The reactions of the gastrointestinal tract are mainly caused by direct stimulation of the smooth muscle as macrolides/azalides are motilin-agonists. The newer derivatives of erythromycin (clarithromycin, azithromycin) are better tolerated by the stomach than the classical macrolide antibiotic [16]. 

Macrolides can cause allergic reactions. Such reactions are much less common than after the administration of penicillins or other beta-lactam antibiotics.

Reversible cases of hearing loss have been described after high doses of erythromycin (intravenous administration!). Cardiotoxic effects may also occur after macrolides. They cause a QTc prolongation and there is the possibility of serious arrhythmias (torsades de pointes) [17].

Interactions have long been known between erythromycin/clarithromycin and many other drugs metabolized by cytochrome P450-dependent monooxygenases (such as CYP3A). Phase I metabolism of carbamazepine, glucocorticoids, terfenadine, theophylline, ciclosporin and many other drugs is inhibited by macrolides. Especially in the case of substances that can lead to QTc prolongation (for example, terfenadine, pimozide), there is an increased risk of torsades de pointes under combined administration. Based on past experience, the azalide azithromycin is not expected to inhibit the metabolizing enzymes. Case reports of interactions with digitalis glycosides are common to all macrolides/azalides – apparently these are caused by other mechanisms [18].

### Glycopeptides

There are five glycopeptides available. Within the group, the lipoglycopeptides (telavancin, dalbavancin, oritavancin) can be differentiated from the classical antibiotics of this group (vancomycin, teicoplanin). They differ significantly in terms of their pharmacological properties, dosages and indications. There are therefore also significant differences in their tolerability profiles.

Hypersensitivity reactions may occur in treatment with any glycopeptide [19].

Rapid infusion of vancomycin can cause upper body erythema (red-neck or red-man syndrome), pain and cramping of the chest and back muscles due to the release of mediators. Such reactions are virtually absent in treatment with teicoplanin [19]. The duration of infusion of vancomycin should therefore be at least 60 minutes, (recommended infusion time for telavancin 60 minutes, teicoplanin and dalbavancin 30 minutes, oritavancin 3 hours). 

During treatment with glycopeptides, gastrointestinal disorders such as nausea and vomiting may occur. Vancomycin administration may lead to kidney failure. The risk of nephrotoxic reactions increases with higher doses and concomitant administration of other substances with nephrotoxic potential [20], [21], [22]; see also chapter 3 [23]. Renal side effects were more frequent in telavancin clinical trials than in vancomycin. Reports of temporary or permanent hearing impairment are rare [24].

Blood count disorders are rare after glycopeptide administration (transient neutropenia, thrombocytopenia, eosinophilia). The administration of glycopeptides may cause pain at the site of injection (thrombophlebitis).

### Daptomycin

The tolerability of daptomycin was similar to that of the reference substances during clinical trials [25]. Constipation (6.2%), nausea (5.8%), injection site reactions (5.8%) and headaches (5.4%) were the most common. Daptomycin may cause striated muscle reactions [26], [27]. At a dose of 3 mg/kg body weight every 12 hours, reversible increases in creatine phosphokinase (CPK) levels were frequently observed in an early Phase I study. Corresponding effects occur less frequently in once-daily administration. In addition, elevated levels of transaminases, which are also associated with the effects on skeletal muscle, may also be present in the patients. Regular monitoring of patients for signs of myopathy and control of CPK levels once a week is generally recommended for daptomycin treatment [28].

Due to the predominantly renal elimination of daptomycin, concomitant administration of drugs that reduce renal filtration (non-steroidal anti-inflammatory drugs, COX-2 inhibitors) elevated plasma levels of daptomycin must be expected. Concomitant treatment with drugs capable of inducing myopathy should preferably be suspended during treatment with daptomycin, as in some cases significantly increased CPK levels have been observed and isolated cases of rhabdomyolysis have occurred [29]. If concomitant use is unavoidable, CPK levels should be checked more frequently than once a week and the patient carefully monitored.

### Aminoglycosides

The therapeutic range of aminoglycosides is low. All antibiotics in this group are potentially nephrotoxic and ototoxic. In addition, they can interfere with neuromuscular transmission and are therefore contraindicated in myasthenia gravis [30], [31].

Aminoglycosides damage the hair cells of the inner ear and the cells in the proximal kidney tubule. The antibiotics are actively transported into these cells by megalin/cubilin. They accumulate in the tubule cells and eventually lead to apoptosis and necrosis of the cells. Therefore the risk of toxic damage increases significantly if the treatment lasts more than 8 days or if the patient has already been treated with an aminoglycoside within 6 weeks prior to the start of treatment [32].

When the total daily dose is given in a short infusion, ototoxicity and nephrotoxicity tend to be lower than when administered three times a day. This dosing concept has become established [30], [33] because this mode of administration (once-daily dosing) also appears more favorable as regards antibacterial action.

Vestibular damage (dizziness, nystagmus) and cochlear damage are associated especially with impaired renal function or high doses. Initially there is hearing loss at high frequencies [31].

Allergic reactions to aminoglycosides are rare [30].

### Oxazolidinones (linezolid, tedizolid)

Linezolid is the first oxazolidinone used in treatment of humans. During clinical trials it was as well tolerated compared to the contrasting anti-infective agents. Most notable were gastrointestinal disorders, such as vomiting and mild CNS reactions. Prolonged treatment with linezolid (>2 weeks) led to changes in the blood count (thrombocytopenia, neutropenia, anemia). Weekly blood count checks are therefore generally indicated for treatment with linezolid.

Peripheral neuropathy and/or optic neuropathy, very rarely progressing to loss of vision, have been reported in patients treated with linezolid. These reports were mostly about patients treated for longer than the maximum recommended duration of 28 days. Cases of lactic acidosis also occurred with long-term treatment [34], [35], [36].

Linezolid is an inhibitor of monoamine oxidase. Corresponding interactions with simultaneously administered adrenergic or serotonergic drugs may therefore occur. This may be significant in concomitant treatment with selective serotonin re-uptake inhibitors and other drugs such as tricyclic antidepressants, serotonin 5-HT_1_ receptor agonists (triptans), direct or indirect sympathomimetics (including adrenergic bronchodilators, pseudoephedrine or phenylpropanolamine), vasopressor agents (such as epinephrine or norepinephrine), dopaminergic agents (such as dopamine or dobutamine) as well as pethidine or buspirone. If co-administered, linezolid should not be used [37], [38].

A second oxazolidinone has been commercially available along with tedizolid since 2015. It is currently only approved for skin and soft tissue infections. Treatment duration is limited to 6 days. In clinical trials, tedizolid was clearly better tolerated than linezolid. The most common adverse effects were gastrointestinal disturbances [39]. It should be noted that significantly lower doses of tedizolid (200 mg vs 1,200 mg/day) were given in these studies over a shorter period (6 days vs 10 days). Myelosuppressive effects should be expected only with prolonged treatment or higher dosages.

### Lincosamides (clindamycin)

The most common adverse effect of clindamycin treatment is diarrhea due to impaired physiological intestinal flora (5–20%). Following treatment with clindamycin, severe pseudomembranous enterocolitis may occur through selection of *Clostridium* *difficile* [40]. Occasionally, bilirubin and liver enzyme levels in the blood increase in treatment with clindamycin. Hypersensitivity reactions are relatively rare, hematologic disorders such as thrombocytopenia, leukopenia and others are mainly observed with prolonged clindamycin treatment [17].

### Metronidazole

The most common adverse effects associated with metronidazole treatment are gastrointestinal disturbances, which may manifest as bitter eructations, metallic taste and nausea. Diarrhea rarely occurs [41]. Possible neurological disorders may include headaches, dizziness, ataxia and paresthesia. Reversible peripheral neuropathies may occur with high doses and long-term treatment. Cases of aseptic meningitis have been reported in connection with metronidazole treatment [42]. There is a chance of allergic reactions and haematological disorders [43]. A so-called disulfiram effect with simultaneous alcohol intake has been described but the data is non-conclusive [44].

### Tetracyclines (doxycycline) and glycylcyclines (tigecycline)

For intravenous administration, only doxycycline is available from the tetracycline group. Gastrointestinal disorders are the most common adverse events associated with doxycycline treatment. Nausea, vomiting, diarrhea (rarely pseudomembranous enterocolitis) may occur. Tetracyclines have phototoxic potential. CNS reactions can manifest as headaches, nausea and photophobia. Allergic reactions including anaphylaxis are very rare. Excessive injection can cause dizziness, flushing, redness of the face and collapse. Intravenous administration is associated with local irritation and can cause phlebitis (thrombophlebitis). It should therefore, if possible, be administered orally [45].

Tigecycline was more likely to cause gastrointestinal side effects (such as nausea) than the antibiotics used for comparison in the clinical approval studies [46]. Vomiting occurred in 19% (tigecycline), 14% (imipenem) and 3.6% (vancomycin/aztreonam) patients in the Phase III studies. In contrast, an increase in transaminases was observed more frequently with vancomycin/aztreonam and skin reactions were significantly more frequent than with tigecycline (19.3% vs 10.6%). Treatment was discontinued with approximately equal frequency in all groups due to side effects. Gastrointestinal effects are dose-related and more prevalent in female than in male patients [47].

Co-administration of tigecycline and warfarin increased plasma levels of R- and S-warfarin (AUC) by 68% and 29%, respectively. Although no direct effect on blood coagulation has been observed, a review of INR with co-administration is advised.

### Colistin

Among polymyxins, especially polymyxin E (colistin) has been going through something of a renaissance for some years [48]. The most common adverse reactions associated with the use of colistimethate are neurotoxic symptoms such as paresthesia of the face and mouth as well as headaches and muscle weakness. Pruritus and renal dysfunction are also very common [49]. Nephrotoxic reactions were observed at a rate of about 7–45% in clinical trials but it has to be taken into account that different definitions of “nephrotoxicity” were used and that the data are partly from patients with severe underlying diseases, which makes interpretation of the results difficult [50]. In a group of relatively young, male patients (predominantly without underlying diseases), nearly one in two experienced mild, reversible nephrotoxic reactions using the so-called RIFLE criteria. In 21% of patients, treatment was discontinued because of renal dysfunction [51]. Significant nephrotoxic reactions have been reported in the majority of patients already suffering from renal insufficiency at start of treatment [52]. If kidney function is impaired, the dose should be reduced according to the manufacturer’s instructions.

### Fosfomycin

The most common adverse effects of fosfomycin are reactions of the gastrointestinal tract (nausea, nausea, vomiting, diarrhea) and skin (exanthema). Other side effects such as tiredness, headaches or taste irritations also occur. Rarely or very rarely blood count changes have been observed such as eosinophilia or aplastic anemia. Very rarely there was anaphylactic shock or liver function disorders. Phlebitis at the site of administration is common [53], [54], [55], [56].

The organism acquires 14.5 mmol Na^+^ from 1 g fosfomycin (corresponding to 1.32 g fosfomycin sodium). Therefore, serum electrolytes should be monitored at the recommended doses. This is especially important in patients with cardiac insufficiency, edema or secondary hyperaldosteronism. The sodium intake associated with fosfomycin application may also cause potassium losses by increasing potassium excretion. Potassium replacement may therefore be required to avoid hypokalaemia.

### Rifampicin

Gastrointestinal intolerance reactions are commonly seen in treatment with rifampicin. They manifest as loss of appetite, stomach pain, nausea, vomiting, meteorism and diarrhea. There are rare reports of pancreatitis [57].

Hypersensitivity reactions are common [58]. The most common manifestations are fever, erythema exudativum multiforme, pruritus and urticaria. Rarely severe reactions, such as respiratory distress, pulmonary edema, other edema and shock, have been observed. Very rarely a lupus-like syndrome has been described with fever, weakness, muscle and joint pain and the appearance of antinuclear antibodies.

Side effects of rifampicin on the liver are common to very common and are mainly manifested as elevations in transaminases, alkaline phosphatase, gamma-glutamyltransferase, and less commonly serum bilirubin. The values often return to normal despite the continuation of treatment [59].

Visual disturbances, visual loss and optic neuritis may also occur as adverse effects.

A limited and harmless brownish-red discoloration of the tear fluid is due to the intrinsic color of the drug.

In rare cases, rifampicin is associated with eosinophilia, leukopenia, granulocytopenia, thrombocytopenia, thrombocytopenic purpura, hypoprothrombinemia or hemolytic anemia.

Rifampicin is a potent inducer of cytochrome enzymes, Phase II enzymes and transport proteins. For example it causes a clear induction of the cytochromes CYP3A4, 1A2, 2C9, 2C8 and 2C18/19 in the intestinal epithelium and in the liver and can thus accelerate the metabolism of other drugs. It has an inhibitory effect on N-acetyltransferases. Similarly, transport proteins for organic anions (OATP2) are inhibited. In view of the complex and diverse influences on the pharmacokinetically relevant metabolization and transport systems, the clinical physicians should expect an impact on the pharmacokinetics of other concomitant drugs with each therapeutic use of rifampicin [60].

## Antibiotics in pregnancy

Treatment with antibiotics during pregnancy is often unavoidable but the data on benefits and risks is completely inadequate. Individual studies are usually not comprehensive enough to derive clear recommendations on the preferred treatment and statements on possible risks for the unborn child are not possible given the low numbers [61].

The data for the use of widespread anti-infectives is not clear even after decades of use and the assessment is often based on the “experience” of the respective prescribers. Given the principles of evidence-based medicine which are now standard, the assessment of benefits and risks during pregnancy sits on completely inadequate foundations.

Unfortunately, only a sketchy description of the potential risks to embryos and fetuses associated with the use of anti-infective agents during pregnancy is possible by dividing them into different risk classes. The classification in Table 1 (Tab. 1) corresponds to the categories of the US FDA that were customary until recently and allows a rough orientation. However, these categories have not been used by the FDA since 2015 [62].

The Pharmacovigilance Center for Embryo Toxicology at the Institute of Clinical Pharmacology and Toxicology of the Charité in Berlin publishes detailed information on the tolerability of the most important drugs in the various stages of pregnancy and during lactation on Embryotox (http://www.embryotox.de/). The site is freely accessible and also available as an app.

## Note

This is the fourth chapter of the guideline “Calculated initial parenteral treatment of bacterial infections in adults – update 2018” in the 2^nd^ updated version. The German guideline by the Paul-Ehrlich-Gesellschaft für Chemotherapie e.V. (PEG) has been translated to address an international audience.

## Competing interests

The authors declare that they have no competing interests.

## Figures and Tables

**Table 1:**
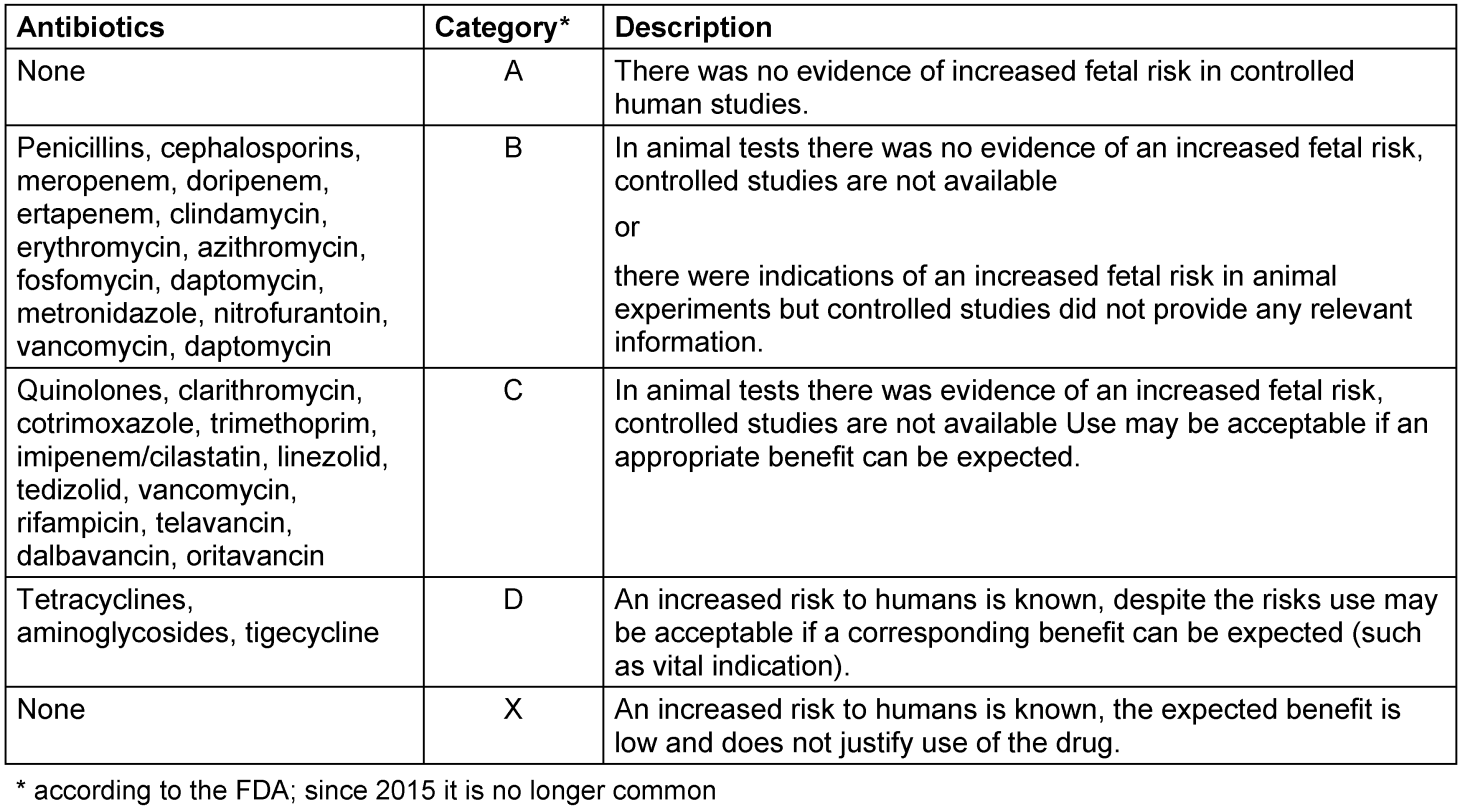
Embryo-fetal risks when using antibiotics
